# A post hoc analysis of the effect of viloxazine extended-release capsules on learning and school problems in children and adolescents with attention-deficit/hyperactivity disorder

**DOI:** 10.1007/s00787-021-01877-5

**Published:** 2021-09-28

**Authors:** Stephen V. Faraone, Roberto Gomeni, Joseph T. Hull, Gregory D. Busse, Zare Melyan, Jonathan Rubin, Azmi Nasser

**Affiliations:** 1 Departments of Psychiatry and of Neuroscience and Physiology, SUNY Upstate Medical University, Syracuse, NY USA; 2 Pharmacometrica, Lieu-dit Longcol, La Fouillade, France; 3 Supernus Pharmaceuticals, Inc., 9715 Key West Ave, Rockville, MD 20850 USA

**Keywords:** SPN-812, Viloxazine, Qelbree, ADHD, Learning, School

## Abstract

Improvement in attention-deficit/hyperactivity disorder (ADHD) symptoms vs. placebo was reported in a series of pediatric clinical trials of viloxazine extended-release capsules (viloxazine ER; Qelbree™). This post hoc analysis of those studies evaluated the effect of viloxazine ER on learning and school problems (LSPs). We used data from four Phase 3 placebo-controlled trials of 100–600 mg/day viloxazine ER (*N* = 1354; 6–17 years of age). LSPs were evaluated using the School domain of the Weiss Functional Impairment Rating Scale-Parent Report (WFIRS-P-S) and the Learning Problems content scale of the Conners 3rd Edition-Parent Short Form (C3PS-LP) at baseline and end of study (≥ Week 6). ADHD symptoms were assessed weekly using the ADHD Rating Scale 5th Edition. The analyses were performed using the general linear mixed model with participant as a random effect. The responder analyses were performed using the Chi-square test. Viloxazine ER demonstrated significantly greater improvements in WFIRS-P-S (*p* < 0.0001) and C3PS-LP (*p* = 0.0113) scores vs. placebo. The response rate for the WFIRS-P-S was significantly greater for viloxazine ER vs. placebo (*p* = 0.001), and the number needed to treat (NNT) was 10.3 (effect size 0.7). Conversely, response rates for C3PS-LP did not differ between groups (*p* = 0.9069). In addition to ADHD symptoms improvement demonstrated in previous studies, viloxazine ER significantly reduced LSPs in pediatric subjects with ADHD. The responder analyses and NNT estimates indicate that a substantial number of children and adolescents with ADHD treated with viloxazine ER improved in clinically assessed LSPs.

## Introduction

It is well known that the symptoms of attention-deficit/hyperactivity disorder (ADHD) affect learning ability and school achievements in individuals with ADHD [1]. In a meta-analysis of 72 studies of children, adolescents, college students, and adults with ADHD, patients with ADHD had lower academic achievement scores compared with typical controls [2]. Consistent with these findings, a study of nearly 30,000 adults found that those with ADHD were twice as likely not to have graduated from high school on time, even after accounting for psychiatric comorbidities [3]. The analysis of education outcome data from three large case–control studies demonstrated that ADHD was an independent major risk factor for grade retention and early termination of education (after adjusting for social class, IQ, and learning disability) [4]. An 8-year longitudinal French study of 1264 subjects found that individuals with inattention and hyperactivity symptoms were less than half as likely to graduate from secondary school [5]. According to school staff reports, over 35% of 543 high-school students with ADHD attended special education classes [6]. In a population-based study of Scottish children, those who were receiving medication for ADHD (primarily stimulants) were at threefold increased risk for low educational achievement, twofold increased risk for dropping out of school before age 16, and eightfold increased risk for needing special educational services. These associations remained strong and significant even after adjusting for socioeconomic confounders and other psychiatric conditions [7].

A few studies have assessed the effects of medications used to treat ADHD on learning and school problems (LSPs) using randomized controlled clinical trials (RCTs) and naturalistic designs. A 9-week, double-blinded RCT compared 101 atomoxetine (ATX)-treated and 99 lisdexamfetamine dimesylate (LDX)-treated subjects with ADHD using the LSP scale of the Weiss Functional Impairment Rating Scale-Parent Report (WFIRS-P) [8]. LDX was superior to ATX for reducing LSPs, with a statistically significant standardized mean difference of 0.43. A 24-week, open-label clinical trial of 168 children and adolescents compared osmotic-release oral system methylphenidate (OROS-MPH) with ATX [9]. At week 24, OROS-MPH improved school functions with a Cohen *d* of − 0.82 for parent-reported outcomes and − 0.66 for self-reported outcomes. For ATX, the Cohen *d* was − 0.62 for parent-reported outcomes and − 0.34 for self-reported outcomes. The differences between the drugs were not significant. In contrast to these results, ATX did not improve teacher ratings of academic productivity in a placebo-controlled RCT of 153 youth [10].

A post hoc analysis of an 8-week, double-blind, placebo-controlled, dose optimization study of 333 children with ADHD (6–12 years of age) assessed the effect of guanfacine extended release (GXR) on LSPs [11]. The study found that GXR treatment led to significant improvements in the WFIRS-P LSP scores vs. placebo. In contrast to these results, a 13-week, double-blind, placebo-controlled RCT found that once-daily GXR (1–7 mg per day) did not improve the WFIRS-P LSP score in adolescents (13–17 years of age) with ADHD at Week 13 [12]. Coghill et al. [13] reviewed two Phase 3 clinical trials of LDX and one of GXR and concluded that LDX was more effective than GXR in improving the WFIRS-P LSP score. LDX, however, was not more effective than OROS-MPH in improving the same score [13, 14].

Using the Swedish population registers, a study of over 650,000 students examined the effects of ADHD medications (primarily stimulants) on school performance. It found that three months of treatment led to a nine-point increase in a summary score of school tests [15]. The treatment was also associated with an increase in the probability of completing upper secondary school by two-thirds [15]. In another large population-based study of long-term school outcomes, subjects with ADHD treated with stimulants were 1.8 times less likely to be held back a grade compared with non-treated subjects. The proportion of school dropouts, however, was similar between treated and non-treated cases [16].

Viloxazine extended-release capsules (viloxazine ER; Qelbree™), a novel non-stimulant medication, has been approved by the U.S. Food and Drug Administration for the treatment of ADHD in children and adolescents (ages 6–17 years). Viloxazine is a selective norepinephrine reuptake inhibitor. Preclinical studies have also demonstrated activity in the serotonergic system in the prefrontal cortex, although the translation of these observations into humans remains to be elucidated [17]. Recently, completed Phase 3 clinical trials have demonstrated efficacy of viloxazine ER in improving ADHD symptoms vs. placebo in children (6–11 years of age) and adolescents (12–17 years of age) with ADHD [18–21]. In a post hoc analysis of these four Phase 3 pediatric trials, viloxazine ER treatment was also associated with statistically significant improvements in executive function [22]; deficits in executive function have long been associated with poor learning and school related outcomes in ADHD [1, 23–25]. In another post hoc analysis evaluating the effect of viloxazine ER on inattention and hyperactivity/impulsivity-associated functional impairments, the improvements in mean change from baseline (CFB) were observed in all three learning and school related impairment items of ADHD Rating Scale 5th Edition (ADHD-RS-5): completing/returning homework, performing academically at school, and controlling behavior at school [26]. The current post hoc analysis evaluates the degree to which viloxazine ER improves parent-reported assessments of LSPs in pediatric subjects with ADHD using the School domain of the Weiss Functional Impairment Rating Scale-Parent Report (WFIRS-P-S) and the Learning Problems content scale of the Conners 3rd Edition-Parent Short Form (C3PS-LP) scores.

## Methods

### Data description

We used data from four Phase 3, randomized, double-blind, three-arm, placebo-controlled clinical trials of viloxazine ER in children and adolescents (6–17 years of age) with ADHD (Table 1) [18–21].

**Table 1:** Overview of phase 3 randomized controlled trials providing data

Study	Age group (yrs)	Weeks (*T* + *M*)	N					Viloxazine ER (mg/day)	*p* value on primary endpoint^b^
			Analysis population			Treatment arm			
			Randomized	Safety	ITT	Viloxazine ER^a^	PBO^a^		
P301	6–11	6 (1 + 5)	477	474	460	305	155	100	0.0004
								200	< 0.0001
P302	12–17	6 (1 + 5)	310	308	301	197	104	200	0.0232
								400	0.0091
P303	6–11	8 (≤ 3 + 5)	313	310	301	204	97	200	0.0038
								400	0.0063
P304	12–17	7 (≤ 2 + 5)	297	296	292	196	96	400	0.0082
								600	0.0712^c^

*CFB* change from baseline, *EOS* end of study, *ITT* intent-to-treat, *M* maintenance, *N* number of subjects, *PBO* placebo, *T* titration, *viloxazine ER* viloxazine extended-release capsules

^a^*N* based on ITT population

^b^CFB in the ADHD-RS-5 Total score at EOS (viloxazine ER vs. placebo)

^c^Although the difference vs. placebo was < 0.05 for the 400 mg group, neither dose could be considered superior to placebo due to the use of statistical method of sequential testing [21]

In each study, informed consent/assent forms were signed as applicable. Each study protocol was approved by Advarra Institutional Review Board (IRB) and conducted in accordance with the Helsinki Declaration and the International Council for Harmonisation Note for Guidance on Good Clinical Practice. All versions of the informed consent/assent form were reviewed and approved by the IRB.

Eligibility was determined based on the following inclusion criteria: diagnosis of ADHD based on DSM-5 criteria confirmed by the Mini International Neuropsychiatric Interview for Children and Adolescents, ADHD-RS-5 Total score ≥ 28, and Clinical Global Impression-Severity of Illness score ≥ 4 [18]. Key exclusion criteria were: major psychiatric disorder or neurological disorder (excluding oppositional defiant disorder, or major depressive disorder if the subject was free of major depressive episodes both currently and for the 6 months prior to screening), a history of allergic reaction to viloxazine or its excipients, any food allergy or intolerance that contraindicated trial participation, suicidal ideation, history of seizures, significant systemic disease, or body mass index > 95th percentile for the appropriate age and sex [18]. Children and adolescents had to weigh ≥ 20 kg and ≥ 35 kg, respectively.

After a screening period of up to 28 days, including a 7-day washout period of medications prohibited by the study protocol, eligible subjects were randomized in a 1:1:1 ratio to receive one of the two doses of viloxazine ER or placebo (Table 1). Subjects were instructed to take the study medication capsules daily by mouth in the morning, with or without food, throughout the treatment period. The viloxazine ER and placebo capsules were identical in appearance. If necessary, the subject’s parent(s) or legal guardian(s) could open the capsules and sprinkle the contents over a spoonful of soft food (e.g., applesauce) [18].

Subjects were assessed weekly for efficacy and safety until end of study (EOS) or early termination. ADHD-RS-5 was measured at screening, baseline, and weekly at post-baseline study visits until EOS or early termination. The WFIRS-P and C3PS were administered at baseline and EOS. The WFIRS-P assesses functionality, specifically to what degree a subject’s behavior and emotional problems affect their interactions and ability to accomplish daily tasks. It has 50 items grouped into six domains scored using a 4-point Likert scale. The domains are family, school, life skills, self-concept, social activities, and risky activities [27, 28]. The parent/caregiver is asked to rate the child on the 50 items based on the child’s presentation over the previous month. The C3PS assesses behavior across six content scales scored on a 4-point Likert scale. The content items are inattention, hyperactivity, learning problems, executive functioning, defiance/aggression, and peer relations [29]. The parent/caregiver is asked to rate the child on 43 of 45 items based on the child’s presentation during the previous month.

LSPs were assessed with WFIRS-P-S average score and C3PS-LP *T*-score.

### Data analyses

The analyses used the general linear mixed effect (GLME) model (SAS version 9.4). The first analysis included all subjects who had WFIRS-P-S/C3PS-LP scores at EOS. This score was used as a dependent variable and the following fixed effects were used as independent variables: WFIRS-P-S/C3PS-LP at baseline, group (drug vs. placebo), sex, and study site. To find out if the means between two groups were significantly different, the *F* test was used. The *F* test is a statistical test, in which the ratio of two variances has an F-distribution under the null hypothesis. The degrees of freedom of the numerator and denominator of the ratio define the reference value of the F-distribution. Since some subjects did not exhibit LSPs at baseline, the GLME model analysis was run again for a subset of individuals who had a WFIRS-P-S average score greater than the median WFIRS-P-S average score (1.4) at baseline and a C3PS-LP *T*-score greater than 70 at baseline (*very elevated*).

The responder rates for the ADHD-RS-5 Total score, WFIRS-P-S average score, and C3PS-LP *T*-score were analyzed using the Chi-Square test. The response rate was based on the percentage of subjects who are defined as responders. An ADHD-RS-5 responder was defined as a subject who had 50% or more reduction in ADHD-RS-5 Total score from baseline to Week 6 (common efficacy endpoint for all studies). A WFIRS-P-S responder was defined as a subject who had a 50% or greater reduction in WFIRS-P-S average score from baseline to EOS (Week 6 or later, study-specific; see Table 1). A C3PS-LP responder was defined as a subject who had C3PS-LP *T*-score greater than 70 (*very elevated*) [29] at baseline and less than 65 (above a 65 score is considered elevated) [29] at EOS. A cutoff *T*-score of 70 was chosen because it was two standard deviations from the population mean, which is a standard method of defining severe impairment for *T*-scores [29].

## Results

Table 2 lists the mean (± SD) WFIRS-P-S average score and C3PS-LP *T*-score at baseline and EOS for subjects treated with placebo and viloxazine ER. The mean WFIRS-P-S average score at baseline was within the severe range of impairment [28] for both treatment groups.

**Table 2:** Learning and school problems observed scores at baseline and endpoint

Treatment	Viloxazine ER	Placebo
*N*	760	394
WFIRS-P-S average score at baseline, mean ± SD	1.48 ± 0.72	1.40 ± 0.71
WFIRS-P-S average score at EOS, mean ± SD	0.98 ± 0.71	1.10 ± 0.74
C3PS-LP *T*-score at baseline, mean ± SD	70.56 ± 13.34	70.18 ± 13.55
C3PS-LP *T*-score at EOS, mean ± SD	64.16 ± 13.62	65.63 ± 14.19

*C3PS-LP* Conners 3rd Edition-Parent Short Form’s Learning Problems content scale, *EOS* end of study, *N* number of subjects, *SD* standard deviation, *viloxazine ER* viloxazine extended-release capsules, *WFIRS-P-S* Weiss Functional Impairment Rating Scale-Parent Report’s School domain

There was a significant effect of the WFIRS-P-S baseline score (*F*_1, 1125_ = 363.6, *p* < 0.0001) and of the viloxazine ER treatment group (*F*_1, 1125_ = 18.6, *p* < 0.0001). A significant effect was also observed for sex (*F*_1, 1125_ = 5.0, *p* = 0.0260), but not for age. The CFB in the WFIRS-P-S average score (least squares means ± SD) was greater in the viloxazine ER group (0.49 ± 0.79) compared with placebo (0.33 ± 1.06), and the difference (0.17 ± 1.30) was statistically significant (*p* < 0.0001) (Table 3).

**Table 3:** Change from baseline in the learning and school problems scores (LS Means estimated in the general linear mixed effect model)

Change from baseline in the WFIRS-P-S average score, LS Means				
Group	Treatment	CFB	SD	*p* value
All subjects				
	Placebo	0.33	1.06	
	Viloxazine ER	0.49	0.79	
	Viloxazine ER − placebo	0.17	1.30	< 0.0001
Subjects with baseline average score > 1.4				
	Placebo	0.58	1.32	
	Viloxazine ER	0.82	0.91	
	Viloxazine ER − placebo	0.24	1.60	0.0006

Change from baseline in the C3PS-LP T-score, LS Means				
	Treatment	CFB	SD	*p* value
All subjects				
	Placebo	4.63	18.49	
	Viloxazine ER	6.34	13.33	
	Viloxazine ER − placebo	1.71	22.80	0.0113
Subjects with baseline *T*-score > 70				
	Placebo	9.17	20.82	
	Viloxazine ER	10.08	15.02	
	Viloxazine ER − placebo	0.92	25.68	0.3839

*C3PS-LP* Conners 3rd Edition-Parent Short Form’s Learning Problems content scale, *CFB* change from baseline, *LS* least squares, *SD* standard deviation, *viloxazine ER* viloxazine extended-release capsules, *WFIRS-P-S* Weiss Functional Impairment Rating Scale-Parent Report’s School domain

When limiting the analysis to those with a WFIRS-P-S average score greater than the median WFIRS-P-S average score (1.4) at baseline, there was a significant effect of baseline WFIRS-P-S average score (*F*_1, 525_ = 52.5, *p* < 0.0001) and of viloxazine ER CFB at EOS (*F*_1, 525_ = 11.8, *p* = 0.0006). The effects of age and sex were not significant. The CFB in the WFIRS-P-S average score was greater in the viloxazine ER group (0.82 ± 0.91) compared with placebo (0.58 ± 1.32), and the difference (0.24 ± 1.60) was statistically significant (*p* = 0.0006).

The mean baseline C3PS-LP *T*-score was within the *very elevated* range of severity [29] for both treatment groups (Table 2). There was a significant effect of the C3PS-LP baseline score (*F*_1, 1149_ = 227.5, *p* < 0.0001) and of the viloxazine ER treatment group (*F*_1, 1149_ = 6.4, *p* = 0.0113). The effects of age and sex as sources of variability were not statistically significant in this analysis. The CFB in the C3PS-LP *T*-score was greater in the viloxazine ER group (6.34 ± 13.33) compared with placebo (4.63 ± 18.49), and the difference (1.71 ± 22.80) was statistically significant (*p* = 0.0113) (Table 3).

When limiting the analysis to those with a C3PS-LP baseline *T*-score greater than 70, there was a significant effect of baseline C3PS-LP *T*-score (*F*_1, 593_ = 23.0, *p* < 0.0001), but not treatment group (*F*_1, 593_ = 0.8, *p* = 0.3839). The effects of age and sex were not statistically significant. The CFB in the C3PS-LP *T*-score was greater in the viloxazine ER group (10.08 ± 15.02) compared with placebo (9.17 ± 20.82), but the difference (0.92 ± 25.68) was not statistically significant (*p* = 0.3839).

The WFIRS-P-S response rate was higher in the viloxazine ER group (36.6%) compared with placebo (26.9%), the difference was statistically significant ($X_{1}^{2}$ = 10.9, *p* = 0.001), and the number needed to treat (NNT) was 10.3. The difference between C3PS-LP response rate in the viloxazine ER group (14.7%) and placebo (14.5%) was not statistically significant ($X_{1}^{2}$ = 0.014, *p* = 0.9069).

**Fig. 1:**
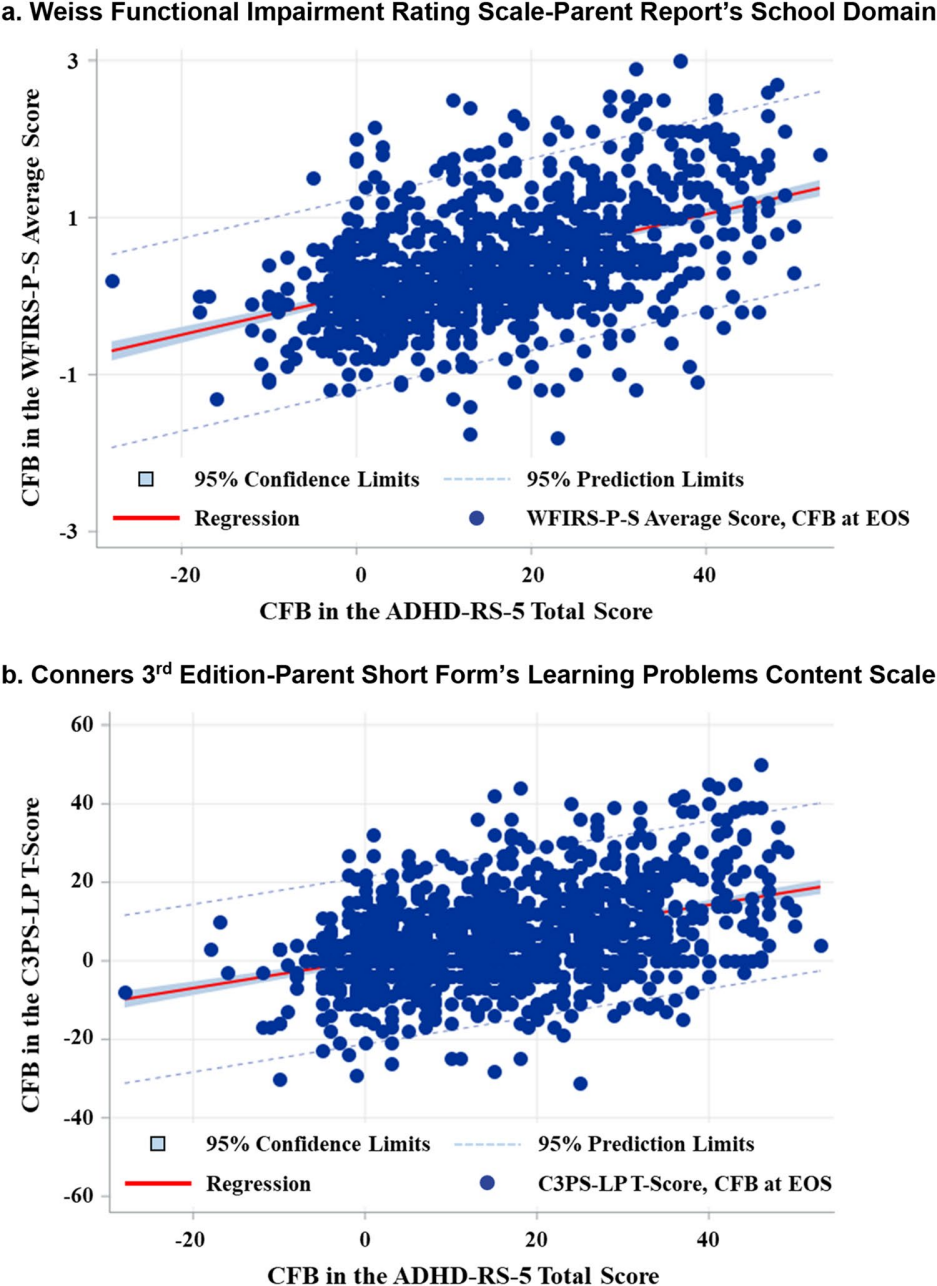
Association between CFB in the Learning and School Problems and CFB in the ADHD-RS-5 with Viloxazine ER Treatment. **a** Weiss Functional Impairment Rating Scale-Parent Report’s School Domain. **b** Conners 3rd Edition-Parent Short Form’s Learning Problems Content Scale. Positive scores denote improvement (C3PS-LP and WFIRS-P-S scores on *y* axis are calculated by subtracting the EOS score from the baseline score; ADHD-RS-5 scores plotted on *x* axis are calculated by subtracting the Week 6 score from the baseline score). *ADHD-RS-5* ADHD Rating Scale 5th Edition, *C3PS-LP* Conners 3rd Edition-Parent Short Form’s Learning Problems content scale, *CFB* change from baseline, *EOS* end of study, *viloxazine ER* viloxazine extended-release capsules, *WFIRS-P-S* Weiss Functional Impairment Rating Scale-Parent Report’s School domain

Among the viloxazine ER-treated subjects, the Pearson’s correlation coefficient (*r*) between the magnitude of response on the WFIRS-P-S average score and the magnitude of ADHD symptom response was 0.49 (*p* < 0.0001). The corresponding Pearson’s correlation coefficient for the C3PS-LP *T*-score and the magnitude of ADHD symptom response was 0.41 (*p* < 0.0001). Figure 1 shows the scatterplots corresponding to these two correlation coefficients.

## Discussion

In previous reports, viloxazine ER was associated with improvements in ADHD symptoms as measured by the ADHD-RS-5 Total scores (Table 1) and Inattention and Hyperactivity/Impulsivity subscale scores [18–21]. All doses of viloxazine ER demonstrated safety and tolerably with low (< 5%) discontinuation rate due to adverse events [18–21]. In this analysis of the effect of viloxazine ER on LSPs in pediatric subjects with ADHD, viloxazine ER significantly reduced LSPs as measured by the WFIRS-P-S domain (*p* < 0.0001) and the C3PS-LP content scale (*p* = 0.0113) scores. Contrary to expectations, the effect of viloxazine ER was not greater when the analysis was limited to subjects with severe levels of LSPs at baseline, possibly because the subjects in the sample had high LSP scores at baseline (Table 2).

The changes in the WFIRS-P-S domain average score were larger and more significant than the changes in the C3PS-LP content scale *T*-score. One potential explanation is that the WFIRS-P-S domain is composed of ten items, whereas the C3PS-LP content scale includes five items. Scales with a greater number of items are usually more reliable and, therefore, are better able to discriminate between groups than are scales with fewer items [30]. Another possible explanation is the content of the individual items. All five items for the C3PS-LP content scale assess academic performance (e.g., "has trouble with reading”) [31]. In contrast, four of ten items for the WFIRS-P-S domain assess academic performance (e.g., "needs tutoring") while the remaining six items assess behavioral problems at school (e.g., "causes problems for the teacher in the classroom") [28]. It seems likely that measures of academic performance would change more slowly than measures of behavioral disturbance. Thus, longer-term trials may be needed to detect more dramatic changes in the C3PS-LP content scale.

Figure 1 shows that, although there is a significant correlation between the change in LSPs and the change in symptoms of ADHD, there are subjects who improve more in one area than the other. For example, some subjects whose C3PS-LP content scale *T*-score improved more than 20 points (two standard deviations), show less than a ten-point improvement in ADHD symptoms (ADHD-RS-5 Total score), while some subjects with a 40-point improvement in ADHD symptoms show little or no improvement in the C3PS-LP content scale *T*-score. This is consistent with the finding that the two change scores share only 16% of their variance. Figure 1a also shows that many subjects achieve large improvements in both the WFIRS-P-S domain average score and ADHD symptoms. For instance, the upper right quadrant shows that, for many subjects, their WFIRS-P-S domain average score improves more than 1.4 points (two standard deviations) and their ADHD symptom score improves 20 or more points. Similar results were observed for the C3PS-LP content scale *T*-score (Fig. 1b).

These results may help clinicians evaluate the impact of viloxazine ER treatment in the context of reducing the core symptoms of ADHD and improving LSPs, which may ultimately lead to better academic performance of the individual. The effect sizes [32] of other medications for ADHD were calculated in long-term studies (e.g., 0.62 for ATX and 0.82 OROS-MPH for parent-reported outcomes were calculated at 24 weeks of treatment) [9]. The present analysis of viloxazine ER used the data from much shorter (6–8 weeks) studies, however, the effect size on WFIRS-P-S was 0.7 (standardized mean difference; all patients). The effect size on C3PS-LP was smaller (0.11), but it is important to note that some subjects did show large improvements in both WFIRS-P-S and C3PS-LP scores. This suggests that the individual approach to evaluating a medication’s effect on LSPs may better assist a clinician in determining the extent of the treatment benefit.

Observations that the improvement in functional impairment is not necessarily associated with the change in ADHD symptoms following a treatment [33, 34] indicate the unmet need for medications that not only lead to symptom improvement, but also improve an individual’s behavior in important functional areas such as LSPs. The effect of viloxazine ER on LSPs extends previous findings of improvements in ADHD symptoms, functional impairments, and executive function deficits with viloxazine ER treatment in pediatric subjects with ADHD [18–20, 22, 26]. The attractiveness of viloxazine ER as a non-stimulant treatment option is further increased by our prior finding that symptom response on ADHD-RS-5 Total score at Week 6 can be predicted, with reasonable accuracy, after 2 weeks of treatment [35].

This work should be evaluated in the context of some limitations. First, because behavioral measures of school functioning were used, our results may not generalize to measures of achievement or academic productivity. One way to evaluate these aspects prospectively would be a classroom study, albeit classroom studies do not reflect real-life scenarios since they only include individuals with ADHD. Moreover, classroom studies are highly controlled with long days of intensive assessments. A second limitation is that, although the data presented here reflect real-life achievements of individuals in their daily school classes, some individuals participated in these clinical trials when they were not attending school (e.g., summer vacation); therefore, added ‘noise’ may have confounded measurement of LSPs. The third limitation is that short-term clinical trials may not have sufficient duration to capture changes in WFIRS-P-S and C3PS-LP scores or if captured, they may not endure. Future studies with a longer-term follow-up time may further elucidate the effect of viloxazine ER on LSPs and evaluate the real-world impact of this effect on overall academic achievement. Finally, the current findings cannot directly compare to those for other medications, given the differences in how response rates were reported (or not reported) across studies. In the future, studies with longer duration and real-world data may provide further insights into long-term academic outcomes after viloxazine ER treatment.

## Conclusion

This post hoc analysis of four Phase 3 pediatric clinical trials of viloxazine ER demonstrated that viloxazine ER significantly reduced parent-reported LSPs vs. placebo. The responder analyses and NNT estimates in this population indicate that a substantial number of children and adolescents with ADHD treated with viloxazine ER can be expected to achieve improvements in clinically assessed LSPs. The results of this analysis are consistent with the data from two other post hoc analyses demonstrating improvement in executive function deficits (measured with C3PS) [22] and improved clinician-rated functional impairments associated with ADHD (measured with the functional impairment scale of the ADHD-RS-5) [26]. Taken together with primary data reported for viloxazine ER, which demonstrated efficacy in reducing ADHD symptoms and a favorable tolerability profile [18–20], these studies suggest viloxazine ER treatment can lead to clinically meaningful improvements in ADHD symptoms and functional behavior in children and adolescents with ADHD.

## Data Availability

Not available.

## Declarations

### Consents

This is a post hoc analysis of integrated data from four clinical studies. In each study, informed consent/assent forms were signed as applicable. Each study protocol was approved by Advarra Institutional Review Board (IRB) and conducted in accordance with the Helsinki Declaration and the International Council for Harmonisation Note for Guidance on Good Clinical Practice. All versions of the informed consent/assent form were reviewed and approved by the IRB.
